# Electrophysiological analysis of signal detection outcomes emphasizes the role of decisional factors in recognition memory

**DOI:** 10.3389/fnhum.2024.1358298

**Published:** 2024-03-20

**Authors:** Stephan Schneider, Sélim Yahia Coll, Armin Schnider, Radek Ptak

**Affiliations:** ^1^Laboratory of Cognitive Neurorehabilitation, Faculty of Medicine, University of Geneva, Geneva, Switzerland; ^2^Department of Clinical Neurosciences, Division of Neurorehabilitation, University Hospitals of Geneva, Geneva, Switzerland; ^3^Department of Clinical Neurosciences, Division of Neurosurgery, University Hospitals of Geneva, Geneva, Switzerland

**Keywords:** recognition memory, decisional processes, event-related potentials, signal detection theory, model-free approach

## Abstract

**Introduction:**

Event-related potential (ERP) studies have identified two time windows associated with recognition memory and interpreted them as reflecting two processes: familiarity and recollection. However, using relatively simple stimuli and achieving high recognition rates, most studies focused on hits and correct rejections. This leaves out some information (misses and false alarms) that according to Signal Detection Theory (SDT) is necessary to understand signal processing.

**Methods:**

We used a difficult visual recognition task with colored pictures of different categories to obtain enough of the four possible SDT outcomes and analyzed them with modern ERP methods.

**Results:**

Non-parametric analysis of these outcomes identified a single time window (470 to 670 ms) which reflected activity within fronto-central and posterior-left clusters of electrodes, indicating differential processing. The posterior-left cluster significantly distinguished all STD outcomes. The fronto-central cluster only distinguished ERPs according to the subject’s response: yes vs. no. Additionally, only electrophysiological activity within the posterior-left cluster correlated with the discrimination index (d’).

**Discussion:**

We show that when all SDT outcomes are examined, ERPs of recognition memory reflect a single-time window that may reveal a bottom-up factor discriminating the history of items (i.e. memory strength), as well as a top-down factor indicating participants’ decision.

## 1 Introduction

The cognitive underpinnings of human recognition memory have been the subject of intense debate centered around two dominant accounts: the dual-process and the single-process model (Mandler, 1980; Yonelinas, 2002; Eichenbaum et al., 2007). The dual-process account posits that recognition implies two sequential operations: familiarity and recollection (Curran, 2000; Ranganath et al., 2004). Familiarity reflects stimulus knowledge that is assumed to be automatic, fast, threshold-based, and devoid of spatio-temporal details (Yonelinas, 1999). In contrast, recollection involves the conscious and deliberate retrieval of specific details or events from memory (Yonelinas, 2002; Brezis et al., 2017). Among the many tasks used to distinguish between the two processes (e.g., Dobbins et al., 2004; Ranganath et al., 2004; Yonelinas et al., 2019) the remember/know (R/K) paradigm is of particular interest as it allows participants to indicate whether they have access to contextual information (remember) or merely know that the item was presented (know; Düzel et al., 1997).

A major support for the dual-process account of recognition memory is provided by studies using event-related potential (ERP) techniques (Curran and Cleary, 2003; Vilberg et al., 2006; Woodruff et al., 2006). A repeated finding of these studies is that R/K judgments are associated with distinct ERP components over mid-frontal electrodes (“know”: FN400 component) and posterior electrodes (“remember”: Late Positive Component, LPC; for a review see Rugg and Curran, 2007). This temporal separation between R/K judgments, which are interpreted as indicators of recollection vs. familiarity, has been replicated numerous times with ERP (Curran and Cleary, 2003; Vilberg et al., 2006; Woodruff et al., 2006) or functional imaging studies (fMRI; Scalici and Caltagirone, 2017). Further investigations have shown that the two ERP components are differentially affected by manipulations of attention (Curran, 2004), confidence levels (Yonelinas, 2001), or memory performance in amnesia (Aly et al., 2010; Addante et al., 2012). R/K judgments identified with fMRI are associated with neural activations in two distinct regions of the frontal cortex. These findings indicate temporal, spatial, and functional differences associated with R/K judgments, which together support dissociated cognitive processes (Hill and Windmann, 2014; Hoppstädter et al., 2015; Andrew Leynes et al., 2019).

**Figure 1:**
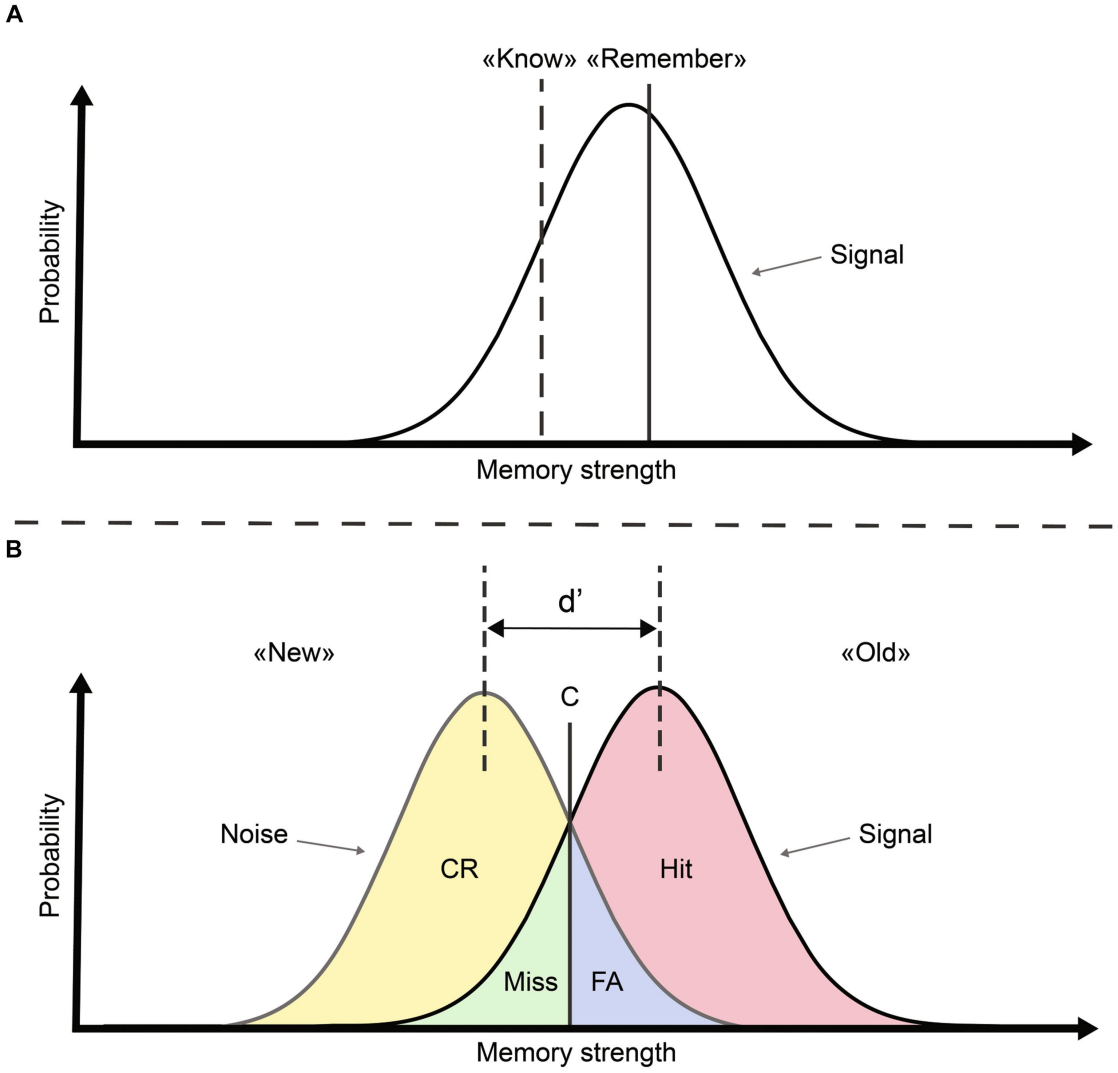
Signal detection theory view of recognition memory modified from Mickes et al. (2007). **(A)** Within a single-process view, familiarity (“know”) and recollection (“remember”) can be characterized as two criteria on the axis representing memory strength. **(B)** SDT conceptualization allows obtaining four outcomes, a decision criterion (C) and a measure of sensitivity (d) based on the spacing between signal and noise distributions.

A less abundant line of research supports the idea that recognition memory relies on a single process. For example, some authors argued that the dissociation between R/K responses is confounded with the strength of the memory trace (Figure 1A; Finnigan et al., 2002; Brezis et al., 2017). According to this proposal, ERP components and time windows might be interpreted as two extremes on the continuum of a single variable: memory strength (Wixted and Stretch, 2004; Wixted, 2009). Some findings also suggest that familiarity and recollection are not stochastically independent regarding their contribution to recognition memory (Moran and Goshen-Gottstein, 2015). The single-process model has stirred considerable debate among scientists, and it is important to note that several subsequent studies have refuted this theory (e.g., Yu and Rugg, 2010; Addante et al., 2012; Addante, 2015). Some reports participating to the debate regarding the cognitive processes underlying recognition memory focused exclusively on correct recognition (i.e., hits and correct rejections; Wais et al., 2008; Hoppstädter et al., 2015), and thus provided arguments based on a biased view of recognition (Wixted, 2009). Several studies considered wrong responses (i.e., misses or false alarms; Curran, 2000; Rugg and Curran, 2007; Wolk et al., 2007; Addante et al., 2023), but a systematic analysis comparing all response types (i.e., correct and wrong responses) in a single analysis that englobes the entire electrode set (instead of focusing on selected electrodes) is missing. Within the framework of Signal Detection Theory (SDT), memory strength elicited by a stimulus represents a value on two overlapping distributions (“signal” and “noise”) that may be attributed to one of four possible outcomes: hit (seen and recognized), miss (seen but not recognized), correct rejection (CR; not seen and not recognized) and false alarm (FA; not seen but recognized). According to SDT, items are judged as “old” if the underlying signal strength exceeds an individual criterion (C; see Figure 1B). The interest in using such an approach is that outcomes can be decomposed according to behavioral response (old vs. new) or ground truth (signal vs. noise). Interestingly, R/K responses can also be considered as criteria on the continuum of memory strength (Donaldson, 1996; see Figure 1A). The R/K procedure is then compatible with an analysis of recognition memory within the SDT framework (Dunn, 2004).

Therefore, the question of interest when considering SDT to understand memory strength and decisional factors as core determinants of recognition memory is whether the four response outcomes generate specific ERP signatures. While some studies have examined memory strength as a moderating factor of ERP components (Brezis et al., 2017), a model-free investigation of ERP signatures of SDT outcomes is lacking. ERP correlates of incorrect responses (misses and false alarms) are less commonly studied, mainly because most previous studies used relatively simple recognition paradigms that did not generate a sufficient number of incorrect answers. For example, several studies used words, non-words, or simple images, which often produce recognition rates exceeding 80% or even 90% (Brady et al., 2008; Brezis et al., 2017; Delorme et al., 2018). These types of stimuli are not sufficiently complex to elicit a substantial number of wrong answers while still maintaining above-chance performance. Some authors have analyzed certain comparisons of responses (Leynes et al., 2005). However, what is missing from these studies is a robust analysis leading to the identification of the regions of interest in terms of temporal windows as well as in spatial/electrodes of maximal discrimination based on the response criterion. With this gap in the literature, the role of response biases and decisional factors in recognition memory remains uncertain. In this study, we applied an old/new task to test recognition memory of natural stimuli that were presented for a limited amount of time to increase the difficulty of the task. The study aimed to analyze old/new ERP data without *a priori* assumptions regarding time windows or regions of interest and without favoring a particular theoretical framework.

## 2 Materials and methods

### 2.1 Participants

Twenty-three healthy participants (14 women, mean age = 25 years, SD = 5) took part in the study after giving their informed written consent. All participants reported no history of psychiatric or neurological disorder, no current use of medication and normal or corrected-to-normal vision. Participants were recruited through flyers around the campus of the University of Geneva. They were remunerated 20 Swiss francs per hour (average remuneration CHF 66). The study was conducted under the approval of Ethics Committee of the Canton of Geneva (approval number 2021-00414). The research reported in this study was performed in accordance with relevant guidelines and regulations. The sample size was determined on the basis of previous ERP studies using an old/new paradigm (Finnigan et al., 2002; MacKenzie and Donaldson, 2007; Hoppstädter et al., 2015).

### 2.2 Stimuli

Stimuli consisted of 720 images, taken from various databases (SwissTopo, Chicago Face Database, THINGS database) and photos^1^ freely available under Creative Commons License. Images were from six categories: landscapes (160 images), buildings (160), objects (160), living (160), neutral human faces rated as “mixed race” (40) and fractals (40). Half of the stimuli of each category were presented on day one and the remaining half on day two. All images were preprocessed by removing any text (e.g., billboards) and people (e.g., workers on a construction site) using Adobe Photoshop (Adobe Inc., 2019). They were then normalized to achieve equal luminosity by scaling each RGB channel to an average value of 127 using a custom MATLAB script (MATLAB, *R2022b*. Natick, Massachusetts: The MathWorks Inc.). Images were cropped to 900 × 900 pixels yielding an image size of 22 × 22, corresponding to 21° at a viewing distance of 60 cm. Stimuli were presented on a EIZO Foris FG2421, 23.5 in the screen with a refresh rate of 60 Hz.

### 2.3 Procedure and task

**Figure 2:**
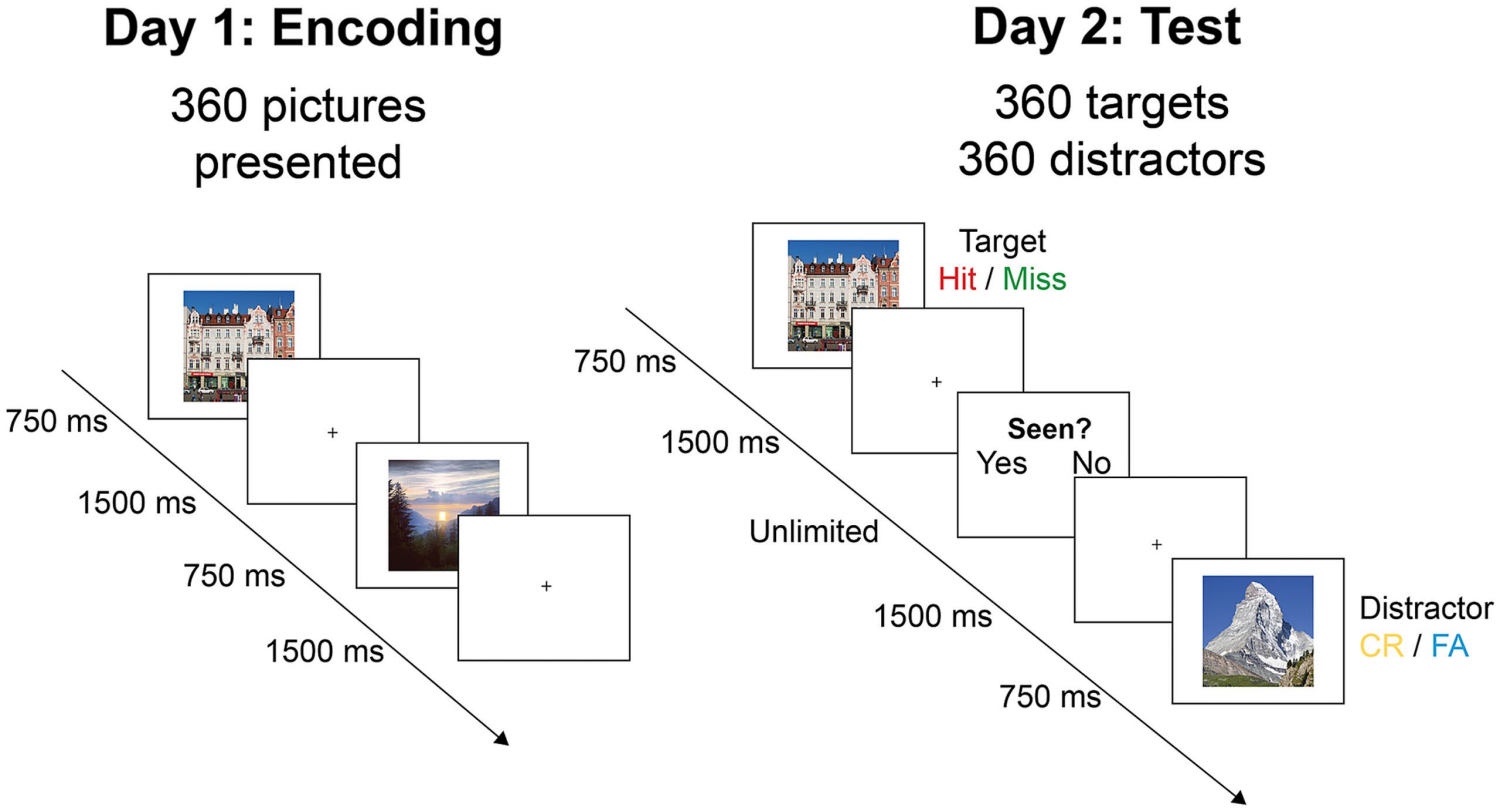
Experimental procedure. On day 1, participants saw 360 images for 750 ms, with the instruction to retain as much information as possible for later recall. On day 2, the same images mixed with 360 foils (distractors) were shown for 1,500 ms. Participants indicated for each image whether they had seen it before by using the keyboard (CR, correct rejection; FA, false alarm).

Participants underwent two experimental sessions separated by 24 h. On the first day (Figure 2; “Day 1”) they were shown half of the images of each category, for a total of 360 items. Participants were instructed to memorize each image for a later recognition test (Figure 2; “Day 2”). Stimuli were displayed for 750 ms, separated by a fixation cross of 1,500 ms, and with randomized presentation order. Day 1 and Day 2 sessions were separated in blocks of approximately 90 trials (4 blocks on Day 1 and 8 blocks on Day 2) lasting about 7 min. Participants could take as much rest as needed between blocks. To ensure attentional engagement during the task, a control task was introduced, consisting of a white arrow appearing at random intervals instead of an image. When this happened, participants were prompted to indicate the direction of the arrow by pressing the corresponding arrow key with their left or right index finger.

On the second day (Figure 2; “Day 2”), participants were instructed to identify the 360 images seen on day one among 720 images (50% old items). Images were shown in randomized presentation order for 750 ms, followed by a fixation cross for 1,500 ms. Following the fixation cross, the words “yes” and “no” appeared on the screen inviting subjects to indicate whether they had seen the image on the day before, by pressing the corresponding arrow key. To mitigate laterality effects, half of the participants answered “yes” with their right index finger and “no” with their left index finger, while for the other half the key-answer mapping was reversed. Once the answer was given, a fixation cross was again displayed for 1,500 ms and a new image appeared.

### 2.4 EEG acquisition and preprocessing

High-density EEG was recorded during the second session (day two) using a 128-electrode set-up (BioSemi Active-Two, V.O.F., Amsterdam, The Netherlands) at a sampling rate of 1,028 Hz. In addition, an electrooculogram (EOG) was recorded using 4 external electrodes for later artifact detection. The EOG electrodes were placed at both lateral canthi for horizontal eye movement and above and below the right eye for vertical movement detection.

Preprocessing was performed with BrainVision Analyzer (version 2.2.0, Brain Products GmbH, Gilching, Germany). After filtering (high-pass: 0.25 Hz, low-pass: 30 Hz, Notch: 50 Hz), data were downsampled to 500 Hz to reduce data volume and increase processing speed. The reference was calculated as the average of all electrodes (Brunet et al., 2011), and electrodes displaying abnormal activity were excluded and interpolated (mean number of interpolated electrodes = 2.61 ± 2.98). To remove artifacts due to blinks or saccades, an ocular ICA was performed with information from the EOG.

ERP epochs were then extracted from −200 to 750 ms post-stimulus onset. A baseline correction was performed using the 200 ms pre-stimulus activity, and artifacts were rejected based on a min/max amplitude criterion of −100 μV/+100 μV (mean number of rejected epochs = 1.02 ± 1.3). ERPs were then labeled with their associated response type (i.e., Hit, Miss, CR or FA) based on participant’s answer. After the pre-processing stage, the mean number of artifact-free trials for the different response types was: Hit =185.22 (median = 187, range = 100–282), Miss = 172.87 (median = 173, range = 74–260), CR = 245.35 (median = 250, range = 160–235) and FA = 112.48 (median = 107, range = 28–199).

### 2.5 Global waveform analysis

**Figure 3:**
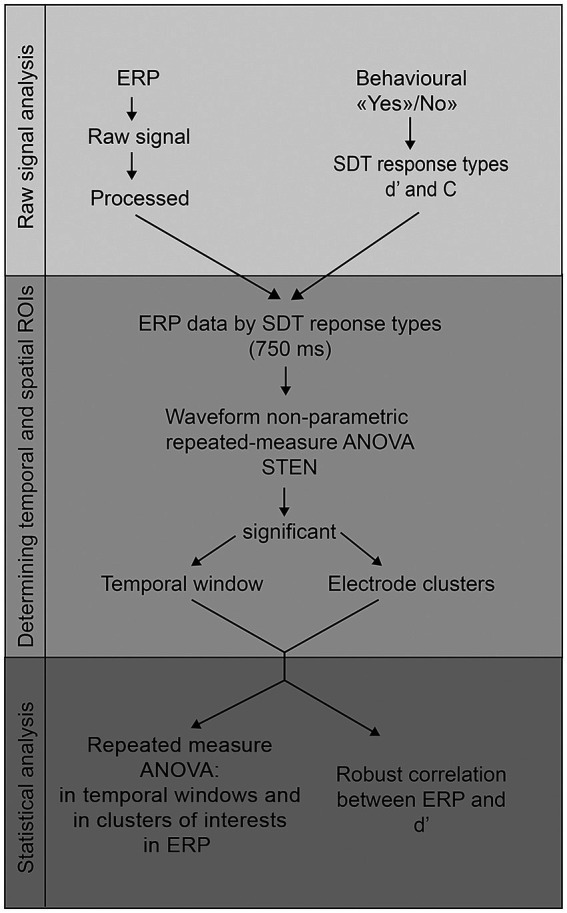
Graphical description of the analysis steps.

To identify the time periods and electrodes of interest without prior assumptions, we adopted a model-free approach of ERP analysis. We used the Statistical Toolbox for Electrical Neuroimaging (STEN) developed by Jean-François Knebel and Michael Notter.^2^ This Python (Python Software Foundation, http://www.python.org) toolbox allows the computation of statistics on several measures of EEG signals with non-parametric waveform repeated-measure analysis of variance (ANOVA), while correcting for family-wise error using the bootstrapping methodology. To summarize the methodology, a *p*-value is calculated for each time point of each electrode in each condition of interest using the bootstrapping method. This involves estimating the sampling distribution of a statistic by drawing samples with replacement from the entire original dataset. The main advantage of this methodology compared to a traditional ERP study is that the selection of electrodes is no longer based on *a priori* assumptions or arbitrary decisions, but solely on statistical criteria, allowing to perform the EEG analysis in a model-free manner. We performed this analysis on all averaged EEG time frames from all participants during the entire period of image presentation (i.e., 0 to 750 ms post-stimulus; Figure 3). The dependent variable was the mean amplitude in microvolts and the fixed factor the SDT response types (Hit vs. Miss vs. CR vs. FA). A bootstrapping with 1,000 iterations was applied to identify significant time frames and electrodes at *p* < 0.05. To eliminate short periods and to identify the region of interest that contains most information, only significant periods longer than 20 ms (i.e., 10 time frames) and only clusters of at least 10 significant non-neighboring electrodes were considered. These criteria are more conservative than in previous studies (e.g., Tautvydaitė et al., 2018), where authors only used time-wise correction. We justify this decision by the hypothesis-free approach of our analysis (Manuel and Schnider, 2016).

Once the clusters identified, the mean ERP value of each SDT outcome was extracted for each participant in each cluster. Using Statistica (version 14.0.0.15, TIBCO software Inc.), a repeated-measure ANOVA with the mean amplitude in microvolts as dependent variable and the SDT response types and Electrode clusters (posterior-left vs. fronto-central) as fixed factor was conducted. In order to take into account the multiple comparison problem, simple effects were performed in Statistica and *p*-values were corrected using the False Discovery Rate methodology (FDR; Benjamini and Yekutieli, 2005).

### 2.6 ERP correlation with d’

A major advantage of SDT is the possibility to express overall memory sensitivity in a single parameter (D-prime or d’) that integrates information from Hit and FA rates based on their standardized difference. The higher the d’ value, the better individuals are able to discriminate signal (targets) from noise (distracters) in a recognition task (Macmillan et al., 2022).

To identify any difference in discrimination performance between the two electrode clusters, we performed correlation analyses between the mean ERP activation in the time window of interest and the d’ of each participant. To control for the impact of outliers we used robust correlations as implemented in the r-skipped correlation in the Robust correlation toolbox (Pernet et al., 2013) on MATLAB (version R2022b, The MathWorks Inc.). The r-skipped correlation attributes a low weight to outlier values and thus provides a more robust computation of the measure of association without loss of power (Pernet et al., 2013).

In order to ensure that the correlations for each cluster were interpretable independently from each other, we performed a comparison of the correlation scores with the Cocor package on R (Diedenhofen and Musch, 2015) using the *z* methodology of Pearson and Filon (1898).

## 3 Results

### 3.1 Behavioral results

The average proportion of correctly recognized items (mean = 0.60, SD = 0.04) was significantly higher than chance for the entire participant group (*t*(22) = 12.5, *p* < 0.001), and for each individual participant. The mean number of responses by SDT response types were as follows: Hit = 186 ± 51.8 (median = 187, range = 100–285); Miss = 174 ± 51.8 (median = 173, range = 75–260); CR = 246 ± 51.5 (median = 252, range = 115–327); FA = 114 ± 51.5 (median = 108, range = 33–245). In terms of SDT parameters, the average d’ was 0.56 (median = 0.55, range = 0.10–1.09) and the decision criterion was 0.23 (median = 0.27, range = −0.46–0.90).

### 3.2 Waveform analysis

#### 3.2.1 Overall results

**Figure 4:**
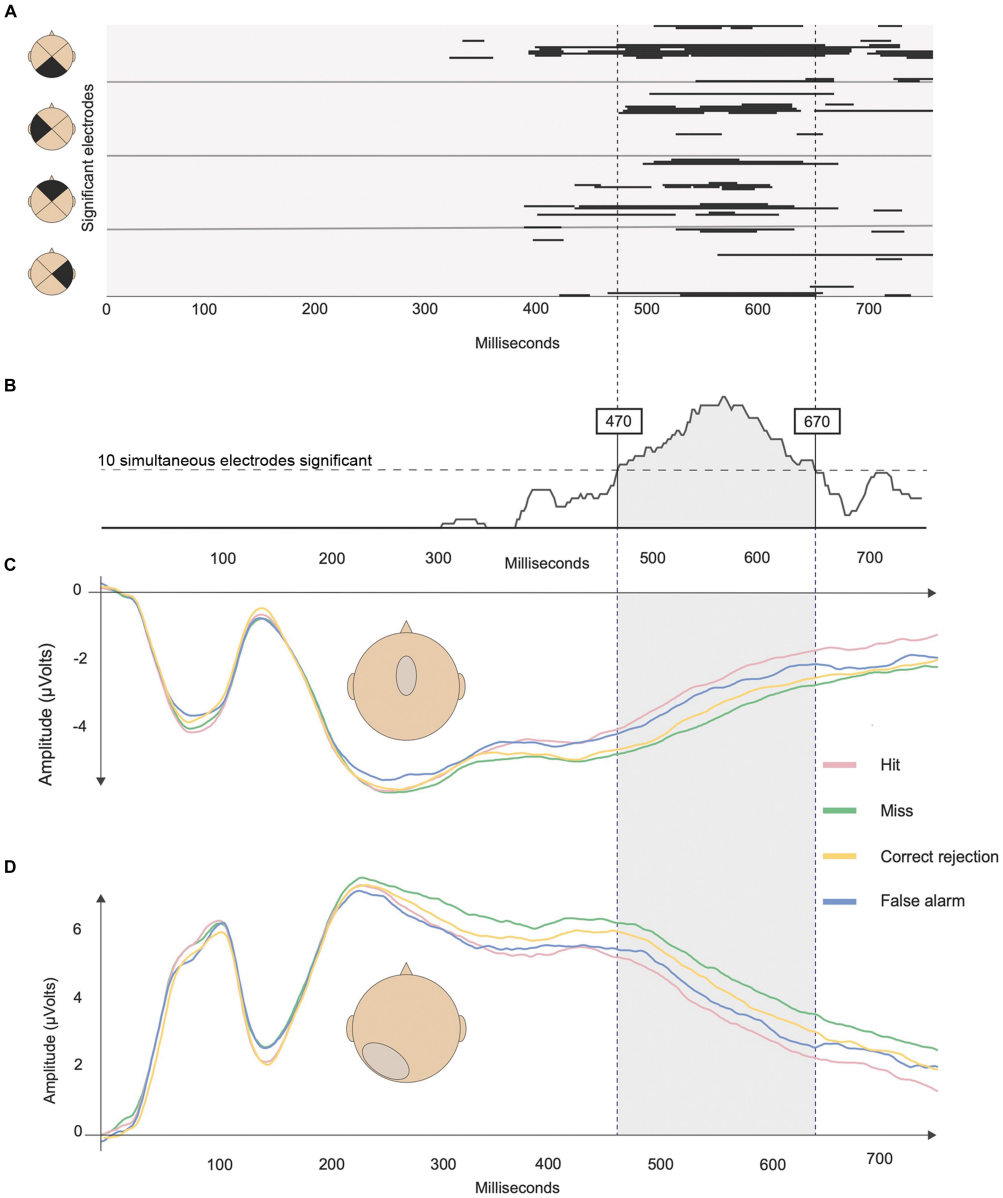
Results of the analysis pipeline to identify the time windows and clusters of significance. **(A)** Output of the non-parametric repeated measure ANOVA on ERP waveforms and the four SDT outcomes. Black lines represent corrected periods of significance (*p* < 0.05 and > 20 ms). Each line represents an electrode from stimulus onset to 750 ms. **(B)** Histogram of the cumulative number of significant electrodes. The horizontal dashed lines represent the minimum criterion of 10 simultaneously significant electrodes. The two vertical lines represent the identified time window of interest between 470 and 670 ms. **(C)** ERPs associated with the four response types at the fronto-central cluster, and **(D)** at the posterior-left cluster.

Figure 4A shows the output of the non-parametric repeated-measure ANOVA computed across the four SDT response types (Hit, Miss, CR and FA) for the entire post-stimulus epoch (0 to 750 ms). The analysis yielded a single-time window between 470 and 670 ms that satisfied the temporal and spatial criteria for significance (Figure 4B).

**Figure 5:**
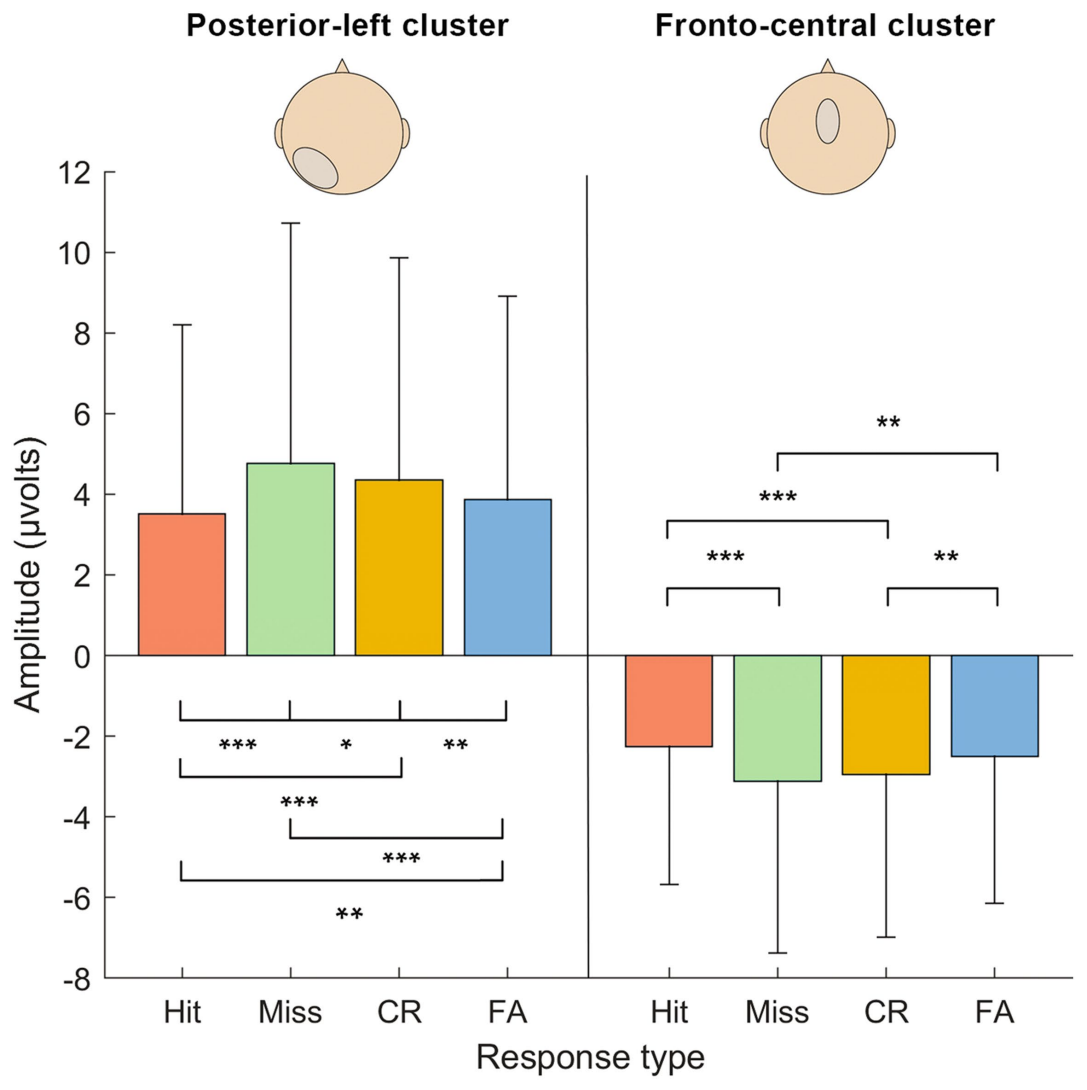
Results of the repeated-measure ANOVA on activation levels (μV) between the response types in each cluster of electrodes (fronto-central and posterior-left) in the identified time window (470–670 ms). Significance levels are marked: *** <0.001, ** <0.01, * <0.5. Errors bars are the confidence intervals at 95%.

Within this time window, two electrode clusters of neighboring electrodes showed significant differences between SDT outcomes (Figures 4, 5). The first cluster was fronto-central around the FC electrode (corresponding roughly to Cz, FCz, C1 and C2 in a 10–10 system) while the second cluster was located in the posterior-left area centered around P3 (corresponding roughly to P1, P3, P5, P7, PO7, PO5, and O1).

#### 3.2.2 Analysis Per electrode cluster

The repeated-measure ANOVA returned significant main effects of Electrode clusters [*F*(1,22) = 42,64, *p* < 0.001, *η_p_*^2^ = 0.660] and SDT response types [*F*(3,66) = 15.56, *p* = 0.022, *η_p_*^2^ = 0.135], as well as a significant interaction between both factors [*F*(3,66) = 27.77, *p* < 0.001, *η_p_*^2^ = 0.558].

To examine the interaction effect further, we performed simple effects between SDT response types within each Electrode cluster (see Table 1). Results revealed that all contrasts between SDT response types were significant for the posterior-left cluster. Concerning the fronto-central cluster, the Hit vs. Miss, Hit vs. CR, Miss vs. FA and CR vs. FA contrasts were significant, but not the Hit vs. FA and Miss vs. CR contrasts.

**Table 1:** Post-hoc results of the repeated measure ANOVA between response types in the two clusters of electrodes identified.

Comparison		Fronto-central	Posterior left
		*p*-values	
Hit	Miss	<0.001	<0.001
	CR	<0.001	<0.001
	FA	0.123	0.006
Miss	CR	0.146	0.010
	FA	0.002	<0.001
CR	FA	0.003	0.002

All *p*-values are corrected using the False Discovery Rate methodology (Benjamini and Yekutieli, 2005). CR, Correct rejections; FA, False alarms.

#### 3.2.3 Correlation between SDT response types and d’

Correlation analyses between d’ and ERP activation in the fronto-central cluster returned non-significant levels of association (r-skipped = 0.18, *t*(91) = 1.73, *p* = 0.866, bootstrapped 90% CI [−0.143, 0.387], no bivariate outliers detected). In contrast, there was a significant negative correlation between ERP activation and d’ in the posterior-left cluster (r-skipped = −0.25, *t*(91) = −2.41, *p* = 0.018, bootstrapped 90% CI [−0.377, −0.025], 4 bivariate outliers detected). The statistical comparison of the two (overlapping) correlations yielded a non-significant result (*z* = 1.73, *p* = 0.084).

## 4 Discussion

Our study focused on SDT parameters to investigate ERP correlates of recognition memory. By manipulating the complexity of the task, we obtained a sufficient number of wrong answers, i.e., FA and misses, to permit analysis of all four possible outcomes, while maintaining performance of all participants above chance. The waveform analysis identified one-time window of interest: 470–670 ms post-stimulus onset, and two clusters of electrodes: fronto-central and posterior-left. Comparisons of ERPs within each cluster showed that all SDT outcomes were well discriminated in the posterior-left cluster. In the fronto-central cluster, however, ERPs reflecting the same behavioral response, i.e., “yes” response (Hit and FA) vs. “no” response (CR and Miss) yielded indistinguishable ERPs even though they had different history: seen (Hit and Miss) vs. unseen (FA and CR). Additionally, we found a significant negative association between the discrimination index (d’) and ERP activation only in the posterior-left cluster. Our findings show that studying SDT outcomes expands our understanding of the electrophysiological correlates of recognition memory.

Behavioral performance in recognition memory often exceeds 80% or even 90% in typical old/new paradigms, indicating that human recognition memory is highly efficient (Manns et al., 2003; Brady et al., 2008; Brezis et al., 2017; Delorme et al., 2018). In our study, recognition performance was considerably lower (about 60%), which might be due to several factors. Many previous ERP studies used simple black and white images or (non-) words (Finnigan et al., 2002; Yu and Rugg, 2010), which have low ecological value and are highly discriminable (Felsen and Dan, 2005; Pinto et al., 2008) as compared to the photographs used in our study. Second, the short presentation time of 750 ms was likely insufficient for a thorough analysis and consequently precluded elaborated encoding of the stimuli. Superficial processing and low discriminability may favor item misses and false positive responses in our task, while previous studies of recognition memory generally only observed few item misses and even fewer FAs (e.g., MacKenzie and Donaldson, 2007; Hoppstädter et al., 2015).

Previous ERP studies testing recognition memory with the R/K paradigm distinguished two time windows that were, respectively, linked to familiarity (at 300–500 ms over fronto-central electrodes) and recollection (400–600 ms over left-parietal electrodes; Rugg and Curran, 2007). In contrast, our ERP analyzes only identified a single time window; that appeared to be temporally coherent with the late ‘parietal’ time window (470–670 ms) identified in previous studies (Curran, 2004; Rugg and Curran, 2007). The crucial question is whether the time window in our study reflects the same underlying cognitive processes related to recognition memory as proposed by other authors. Rugg and Curran (2007) summarized the findings of several studies by concluding that the late parietal component (LPC) is indicative of memory recollection. Their reasoning was based on the observation that the LPC was modulated by successful vs. unsuccessful source judgments or remember vs. know responses. Some also argued that the LPC was not related to response confidence or stimulus probability (Yu and Rugg, 2010). However, some of the findings supporting these conclusions are derived from studies that did not systematically compared correctly and incorrectly classified old and new items. This presents a problem for the interpretation of any electrophysiological component: while missed items may be explained by a failure of familiarity or recollection, it is difficult to explain FAs without recurring to alternative explanations. Examining only correct responses also ignores the fact that recognition memory reflects a decision-making process, particularly when subject’s confidence is weak.

Though the parietal time window reported in previous studies was identified by comparing seen to unseen stimuli, we observed a similar window after integrating all four SDT outcomes in an ANOVA. The slight temporal shift of approximately 70 ms might be explained by increased difficulty of our task, which may have delayed decisions due to uncertainty regarding items held in memory (Murata et al., 2005). We also identified a left-parietal cluster of electrodes that appears to be predominantly activated during this time window. One possible interpretation of this ERP components within our time window could be similar to the LPC described in previous studies, and therefore might reflect related electrophysiological processes. However, as no remember/know ratings were included in the current paradigm a direct comparison between our findings and research using the R/K paradigm is difficult and might be addressed in future studies. This information could be obtained by adding confidence scales to allow more granularity of the old-new paradigm, while still allowing the analysis of all SDT categories.

In contrast to previous work, we also observed a fronto-central electrode cluster that was active during the same time window. Our findings are not compatible with a single-cognitive process taking place in this time window, such as recollection, but rather suggest two distinct contributions to recognition memory. When examining ERP differences across conditions, we found that the posterior-left electrode cluster not only distinguished SDT outcomes according to item history (i.e., whether items had been presented before), but also the judgment of the subject (i.e., whether the subjects thought having seen the item before). In contrast, the fronto-central cluster only distinguished SDT outcomes according to the belief of the subject of having seen the item before. The distinction between item history and the subjective judgment of the observer is important, and can only be captured when all four SDT outcomes are considered.

The main strength of SDT is that it conceives item detection as a decision-making process, whereby subjective assessments, or beliefs, as well as decision criteria, come at play. Focusing only on ERP correlates of correct identifications of old vs. new items may identify electrophysiological processes underlying assumed memory components (such as recollection), but neglects the fact that memory is subject to metacognitive judgments. Our findings therefore open the debate as to which cognitive components are reflected by the identified ERP components. We would argue that in our findings associative strength of memory traces (Brezis et al., 2017) and metacognitive judgments partly dissociate across the posterior-left and the fronto-central electrode cluster. This hypothesis is based on the observation that the latter cluster only exhibited activity differences that could be explained by the type of response (yes vs. no), but not the actual item history. This finding is difficult to reconcile with the proposal that this cluster is specifically linked to familiarity (Hoppstädter et al., 2015), since FA items that were not seen before and should therefore not be familiar were processed similarly to target pictures. Also, interpreting this finding as reflecting the strength of memory traces requires the assumption that some items may have memory traces although they were never seen before. A more plausible possibility is that subjects decide whether they have seen an item before based on a comparison with the item pool, which becomes more difficult the more similar items have been presented.

Our finding thus suggests that the fronto-central cluster is specifically linked to metacognitive processes that guide the decision to produce a yes- or no-response. It is somewhat less straightforward to interpret the meaning of electrophysiological activity extracted from the posterior-left cluster, since this cluster differentiated between item history, but also between behavioral responses. Following our reasoning that responses in a recognition memory task reflect the strength of the memory trace and decisional processes, the posterior-left cluster appears to be a better predictor of performance. This conclusion is also supported by the correlation analysis, which showed that only ERP activity in the posterior-left cluster significantly predicted sensitivity in recognition memory (d’).

To sum up, our study shows that items in a recognition task do not only elicit different responses based on their representation in memory, but are also subject to complex decision processes. Such decision processes might operate on associative information, or on memory strength, which according to some authors may explain results of R/K paradigms within a single-process model (Brezis et al., 2017). However, ERP data extracted from the posterior-left cluster identified in our study can better be reconciled with a decisional process, rather than with the strength of memory representations. Memory strength predicts an arrangement of amplitudes that places hits and misses together (high memory strength), as opposed to CR and FA outcomes (low memory strength). Instead, Figure 5 shows that ERP amplitudes were arranged following the order Miss>CR > FA > Hit, which suggests that outcomes with yes-responses demand lower activations. This pattern suggests that the driving factor in the posterior cluster is not memory strength *per se*, but rather a metacognitive representation motivating a yes- or no-response.

In conclusion, by applying a signal detection framework we observed that the representation of items in memory (i.e., memory strength) as well as decisional processes affect the electrophysiological correlates of recognition memory. Based on our findings, we argue that classifying outcomes according to SDT enhances the possibility to analyze the electrophysiological components of recognition memory. Our study underlines the necessity to consider wrong answers (FA and misses) when analyzing recognition memory, as they may contain important information about the mental processes underlying the functions of human memory.

## Data availability statement

The raw data supporting the conclusions of this article will be made available by the authors, without undue reservation.

## Ethics statement

The studies involving humans were approved by Ethics Committee of the Canton of Geneva (approval number 2021-00414). The studies were conducted in accordance with the local legislation and institutional requirements. The participants provided their written informed consent to participate in this study.

## Author contributions

SS: Conceptualization, Formal analysis, Investigation, Methodology, Visualization, Writing – original draft, Writing – review & editing. SC: Conceptualization, Formal analysis, Investigation, Methodology, Supervision, Visualization, Writing – review & editing. AS: Funding acquisition, Resources, Supervision, Writing – review & editing. RP: Conceptualization, Resources, Supervision, Validation, Writing – review & editing.

## Footnotes

^1^ Unsplash.com

^2^ http://doi.org/10.5281/zenodo.1164038

## References

[ref1] Addante R. J. (2015). A critical role of the human hippocampus in an electrophysiological measure of implicit memory. Neuroimage 109, 515–528. doi: 10.1016/j.neuroimage.2014.12.069, PMID: 25562828 PMC4340755

[ref2] Addante R. J. Lopez-Calderon J. Allen N. Luck C. Muller A. Sirianni L.. (2023). An ERP measure of non-conscious memory reveals dissociable implicit processes in human recognition using an open-source automated analytic pipeline. Psychophysiology 60:e14334. doi: 10.1111/psyp.14334, PMID: 37287106 PMC10524783

[ref3] Addante R. J. Ranganath C. Olichney J. M. Yonelinas A. P. (2012). Neurophysiological evidence for a recollection impairment in amnesia patients that leaves familiarity intact. Neuropsychologia 50, 3004–3014. doi: 10.1016/j.neuropsychologia.2012.07.038, PMID: 22898646 PMC3483383

[ref9001] Adobe Inc (2019). Adobe Photoshop. Available at: https://www.adobe.com/products/photoshop.html.

[ref4] Aly M. Knight R. T. Yonelinas A. P. (2010). Faces are special but not too special: spared face recognition in amnesia is based on familiarity. Neuropsychologia 48, 3941–3948. doi: 10.1016/j.neuropsychologia.2010.09.005, PMID: 20833190 PMC4084520

[ref5] Andrew Leynes P. Batterman A. Abrimian A. (2019). Expectations alter recognition and event-related potentials (ERPs). Brain Cogn. 135:103573. doi: 10.1016/j.bandc.2019.05.011, PMID: 31195236

[ref6] Benjamini Y. Yekutieli D. (2005). False discovery rate–adjusted multiple confidence intervals for selected parameters. J. Am. Stat. Assoc. 100, 71–81. doi: 10.1198/016214504000001907

[ref7] Brady T. F. Konkle T. Alvarez G. A. Oliva A. (2008). Visual long-term memory has a massive storage capacity for object details. Proc. Natl. Acad. Sci. U. S. A. 105, 14325–14329. doi: 10.1073/pnas.0803390105, PMID: 18787113 PMC2533687

[ref8] Brezis N. Bronfman Z. Z. Yovel G. Goshen-Gottstein Y. (2017). The electrophysiological signature of remember–know is confounded with memory strength and cannot be interpreted as evidence for dual-process theory of recognition. J. Cogn. Neurosci. 29, 322–336. doi: 10.1162/jocn_a_01053 27991029

[ref9] Brunet D. Murray M. M. Michel C. M. (2011). Spatiotemporal analysis of multichannel EEG: CARTOOL. Comput. Intell. Neurosci. 2011, 1–15. doi: 10.1155/2011/813870, PMID: 21253358 PMC3022183

[ref10] Curran T. (2000). Brain potentials of recollection and familiarity. Mem. Cognit. 28, 923–938. doi: 10.3758/BF03209340 11105518

[ref11] Curran T. (2004). Effects of attention and confidence on the hypothesized ERP correlates of recollection and familiarity. Neuropsychologia 42, 1088–1106. doi: 10.1016/j.neuropsychologia.2003.12.011, PMID: 15093148

[ref12] Curran T. Cleary A. M. (2003). Using ERPs to dissociate recollection from familiarity in picture recognition. Cogn. Brain Res. 15, 191–205. doi: 10.1016/S0926-6410(02)00192-1, PMID: 12429370

[ref13] Delorme A. Poncet M. Fabre-Thorpe M. (2018). Briefly flashed scenes can be stored in long-term memory. Front. Neurosci. 12:688. doi: 10.3389/fnins.2018.00688, PMID: 30344471 PMC6182062

[ref14] Diedenhofen B. Musch J. (2015). Cocor: a comprehensive solution for the statistical comparison of correlations. PLoS One 10:e0121945. doi: 10.1371/journal.pone.0121945, PMID: 25835001 PMC4383486

[ref15] Dobbins I. G. Kroll N. E. A. Yonelinas A. P. (2004). Dissociating familiarity from recollection using rote rehearsal. Mem. Cognit. 32, 932–944. doi: 10.3758/BF03196871, PMID: 15673181

[ref16] Donaldson W. (1996). The role of decision processes in remembering and knowing. Mem. Cognit. 24, 523–533. doi: 10.3758/BF03200940, PMID: 8757500

[ref17] Dunn J. C. (2004). Remember-know: a matter of confidence. Psychol. Rev. 111, 524–542. doi: 10.1037/0033-295X.111.2.524, PMID: 15065921

[ref18] Düzel E. Yonelinas A. P. Mangun G. R. Heinze H.-J. Tulving E. (1997). Event-related brain potential correlates of two states of conscious awareness in memory. Proc. Natl. Acad. Sci. 94, 5973–5978. doi: 10.1073/pnas.94.11.5973, PMID: 9159185 PMC20891

[ref19] Eichenbaum H. Yonelinas A. P. Ranganath C. (2007). The medial temporal lobe and recognition memory. Annu. Rev. Neurosci. 30, 123–152. doi: 10.1146/annurev.neuro.30.051606.094328, PMID: 17417939 PMC2064941

[ref20] Felsen G. Dan Y. (2005). A natural approach to studying vision. Nat. Neurosci. 8, 1643–1646. doi: 10.1038/nn1608 16306891

[ref21] Finnigan S. J. Humphreys M. S. Dennis S. Geffen G. M. (2002). ERP “old/new” effects: memory strength and decisional factor(s). Neuropsychologia 40, 2288–2304. doi: 10.1016/s0028-3932(02)00113-6, PMID: 12417459

[ref22] Hill H. Windmann S. (2014). Examining event-related potential (ERP) correlates of decision Bias in recognition memory judgments. PLoS One 9:e106411. doi: 10.1371/journal.pone.0106411, PMID: 25264982 PMC4180069

[ref23] Hoppstädter M. Baeuchl C. Diener C. Flor H. Meyer P. (2015). Simultaneous EEG–fMRI reveals brain networks underlying recognition memory ERP old/new effects. Neuroimage 116, 112–122. doi: 10.1016/j.neuroimage.2015.05.026 25988228

[ref24] Leynes P. A. Landau J. Walker J. Addante R. J. (2005). Event-related potential evidence for multiple causes of the revelation effect. Conscious. Cogn. 14, 327–350. doi: 10.1016/j.concog.2004.08.005, PMID: 15950886

[ref25] MacKenzie G. Donaldson D. I. (2007). Dissociating recollection from familiarity: electrophysiological evidence that familiarity for faces is associated with a posterior old/new effect. Neuroimage 36, 454–463. doi: 10.1016/j.neuroimage.2006.12.005, PMID: 17451972

[ref26] Macmillan N. A. Hautus M. J. Creelman C. D. (2022). Detection theory: A user’s guide. 3rd Edn. New York, NY: Routledge.

[ref27] Mandler G. (1980). Recognizing: the judgment of previous occurrence. Psychol. Rev. 87, 252–271. doi: 10.1037/0033-295X.87.3.252

[ref28] Manns J. R. Hopkins R. O. Reed J. M. Kitchener E. G. Squire L. R. (2003). Recognition memory and the human Hippocampus. Neuron 37, 171–180. doi: 10.1016/S0896-6273(02)01147-9 12526782

[ref9003] Mickes L. Wixted J. T. Wais P. E. (2007). A direct test of the unequal-variance signal detection model of recognition memory. Psychon. Bull. Rev., 14, 858–865. 18087950 10.3758/bf03194112

[ref29] Manuel A. L. Schnider A. (2016). Differential processing of immediately repeated verbal and non-verbal stimuli: an evoked-potential study. Eur. J. Neurosci. 43, 89–97. doi: 10.1111/ejn.13114, PMID: 26506905

[ref30] Moran R. Goshen-Gottstein Y. (2015). Old processes, new perspectives: familiarity is correlated with (not independent of) recollection and is more (not equally) variable for targets than for lures. Cogn. Psychol. 79, 40–67. doi: 10.1016/j.cogpsych.2015.01.005, PMID: 25899705

[ref31] Murata A. Uetake A. Takasawa Y. (2005). Evaluation of mental fatigue using feature parameter extracted from event-related potential. Int. J. Ind. Ergon. 35, 761–770. doi: 10.1016/j.ergon.2004.12.003

[ref32] Pearson K. Filon L. N. G. (1898). VII. Mathematical contributions to the theory of evolution. IV. On the probable errors of frequency constants and on the influence of random selection on variation and correlation. Philos. Trans. R. Soc. Lond. Ser. Contain. Pap. Math. Phys 191, 229–311. doi: 10.1098/rsta.1898.0007

[ref33] Pernet C. R. Wilcox R. Rousselet G. A. (2013). Robust correlation analyses: false positive and power validation using a new open source Matlab toolbox. Front. Psychol. 3:606. doi: 10.3389/fpsyg.2012.00606, PMID: 23335907 PMC3541537

[ref34] Pinto N. Cox D. D. DiCarlo J. J. (2008). Why is real-world visual object recognition hard? PLoS Comput. Biol. 4:e27. doi: 10.1371/journal.pcbi.0040027, PMID: 18225950 PMC2211529

[ref35] Ranganath C. Yonelinas A. P. Cohen M. X. Dy C. J. Tom S. M. D’Esposito M. (2004). Dissociable correlates of recollection and familiarity within the medial temporal lobes. Neuropsychologia 42, 2–13. doi: 10.1016/j.neuropsychologia.2003.07.006 14615072

[ref36] Rugg M. D. Curran T. (2007). Event-related potentials and recognition memory. Trends Cogn. Sci. 11, 251–257. doi: 10.1016/j.tics.2007.04.004 17481940

[ref37] Scalici F. Caltagirone C. (2017). The contribution of different prefrontal cortex regions to recollection and familiarity: a review of fMRI data. Neurosci. Biobehav. Rev. 83, 240–251. doi: 10.1016/j.neubiorev.2017.10.017, PMID: 29079492

[ref9002] Tautvydaitė D. Manuel A. L. Nahum L. Adam‐Darqué A. Ptak R. Schnider A. (2018). Absence of an early hippocampal encoding signal after medial temporal lesions: No consequence for the spacing effect. Hippocampus, 29, 587–594. 30421476 10.1002/hipo.23053

[ref38] Vilberg K. L. Moosavi R. F. Rugg M. D. (2006). The relationship between electrophysiological correlates of recollection and amount of information retrieved. Brain Res. 1122, 161–170. doi: 10.1016/j.brainres.2006.09.023, PMID: 17027673 PMC1713226

[ref39] Wais P. E. Mickes L. Wixted J. T. (2008). Remember/know judgments probe degrees of recollection. J. Cogn. Neurosci. 20, 400–405. doi: 10.1162/jocn.2008.20041, PMID: 18004949

[ref40] Wixted J. T. (2009). Remember/know judgments in cognitive neuroscience: an illustration of the underrepresented point of view. Learn. Mem. 16, 406–412. doi: 10.1101/lm.1312809, PMID: 19546229

[ref41] Wixted J. T. Stretch V. (2004). In defense of the signal detection interpretation of remember/know judgments. Psychon. Bull. Rev. 11, 616–641. doi: 10.3758/BF03196616, PMID: 15581116

[ref42] Wolk D. A. Schacter D. L. Lygizos M. Sen N. M. Chong H. Holcomb P. J.. (2007). ERP correlates of remember/know decisions: association with the late posterior negativity. Biol. Psychol. 75, 131–135. doi: 10.1016/j.biopsycho.2007.01.005, PMID: 17329007

[ref43] Woodruff C. C. Hayama H. R. Rugg M. D. (2006). Electrophysiological dissociation of the neural correlates of recollection and familiarity. Brain Res. 1100, 125–135. doi: 10.1016/j.brainres.2006.05.019, PMID: 16774746

[ref44] Yonelinas A. P. (1999). The contribution of recollection and familiarity to recognition and source-memory judgments: a formal dual-process model and an analysis of receiver operating characteristics. J. Exp. Psychol. Learn. Mem. Cogn. 25, 1415–1434. doi: 10.1037/0278-7393.25.6.1415, PMID: 10605829

[ref45] Yonelinas A. P. (2001). Consciousness, control, and confidence: the 3 Cs of recognition memory. J. Exp. Psychol. Gen. 130, 361–379. doi: 10.1037/0096-3445.130.3.361, PMID: 11561915

[ref46] Yonelinas A. P. (2002). The nature of recollection and familiarity: a review of 30 years of research. J. Mem. Lang. 46, 441–517. doi: 10.1006/jmla.2002.2864

[ref47] Yonelinas A. P. Ranganath C. Ekstrom A. D. Wiltgen B. J. (2019). A contextual binding theory of episodic memory: systems consolidation reconsidered. Nat. Rev. Neurosci. 20, 364–375. doi: 10.1038/s41583-019-0150-4, PMID: 30872808 PMC7233541

[ref48] Yu S. S. Rugg M. D. (2010). Dissociation of the electrophysiological correlates of familiarity strength and item repetition. Brain Res. 1320, 74–84. doi: 10.1016/j.brainres.2009.12.071, PMID: 20051232 PMC2826612

